# Virus-induced CXCL10-CXCR3 positive feedback loop via astrocytes is critical for maintaining chronic inflammatory lesions in HAM/TSP

**DOI:** 10.1186/1742-4690-11-S1-O25

**Published:** 2014-01-07

**Authors:** Tomoo Sato, Hitoshi Ando, Utano Tomaru, Mari Yoshida, Atae Utsunomiya, Junji Yamauchi, Natsumi Araya, Naoko Yagishita, Ariella Coler-Reilly, Steven Jacobson, Yoshihisa Yamano

**Affiliations:** 1 Department of Rare Diseases Research, Institute of Medical Science, St Marianna University School of Medicine, Kawasaki, Kanagawa, Japan; 2 Department of Pathology, Hokkaido University Graduate School of Medicine, Sapporo, Hokkaido, Japan; 3 Institute for Medical Science of Aging, Aichi Medical University, Nagakute, Aichi, Japan; 4 Department of Hematology, Imamura Bun-in Hospital, Kagoshima, Kagoshima, Japan; 5 Viral Immunology Section, Neuroimmunology Branch, National Institutes of Health, Bethesda, MD, USA

## 

HTLV-1-associated myelopathy/tropical spastic paraparesis (HAM/TSP) is a debilitating neurologic disorder characterized by chronic inflammation in the spinal cord. However, the precise mechanism by which chronic inflammatory lesions in HAM/TSP are formed and maintained has not been discovered. Since it is believed that chemokines play a central role in lymphocyte recruitment to sites of inflammation, we hypothesized that a positive feedback loop driven by chemokines may be responsible for the chronic inflammation in HAM/TSP. We aimed to determine the identity of these chemokines, where they are produced, and how they drive chronic inflammation in HAM/TSP. We found that HAM/TSP patients have extraordinarily high levels of the chemokine CXCL10 and an abundance of cells expressing the CXCL10-binding receptor CXCR3 in the cerebrospinal fluid. Histological analysis revealed that astrocytes are the main producers of CXCL10 in the spinal cords of HAM/TSP patients. Co-culture of human astrocytoma cells with CD4+ T-cells from HAM/TSP patients revealed that astrocytes produce CXCL10 in response to IFN-γ secreted by CD4+ T-cells. Chemotaxis assays results suggest that CXCL10 induces migration of peripheral blood mononuclear cells to the central nervous system (CNS) and anti-CXCL10 neutralizing antibody can disrupt this migration. In short, HTLV-1-infected cells in the CNS produce IFN-γ that induces astrocytes to secrete CXCL10 that recruits more infected cells to the area via CXCR3, constituting a Th1-centric positive feedback loop that results in chronic inflammation.

